# COVID‐19 Prophylactic Effect of Bromhexine Hydrochloride

**DOI:** 10.1002/iid3.70438

**Published:** 2026-04-20

**Authors:** Vanyo Mitev, Tsanko Mondeshki, Ani Miteva, Konstantin Tachkov, Violeta Dimitrova

**Affiliations:** ^1^ Research Institute of Innovative Medical Science, Medical University‐Sofia Sofia Bulgaria; ^2^ Department of Medical Chemistry and Biochemistry, Medical Faculty Medical University‐Sofia Sofia Bulgaria; ^3^ Department of Propaedeutics of Internal Diseases University Hospital Alexandrovska, Medical University‐Sofia Sofia Bulgaria; ^4^ Department of Bioethics Faculty of Public Health, Medical University‐Sofia Sofia Bulgaria; ^5^ Department of Social Pharmacy and Pharmaco‐economics, Faculty of Pharmacy Medical University‐Sofia Sofia Bulgaria

**Keywords:** Bromhexine, COVID‐19, Prophylactics, TMPRSS2

## Abstract

**Introduction:**

Despite extensive efforts to identify effective treatments for COVID‐19, preventive strategies remain essential. Bromhexine hydrochloride (BRH), an over‐the‐counter mucolytic with potential TMPRSS2 inhibition, may reduce viral entry and thus serve as a prophylactic agent.

**Methods:**

A retrospective questionnaire‐based study included 125 individuals reporting prophylactic BRH use. Demographic and clinical data were collected. COVID‐19 infection rates before and after BRH use were compared. Subgroup analyses were performed by vaccination status, prior infection, and duration of prophylaxis.

**Results:**

Participants had a mean age of 56.5 years and a mean BMI of 25.6; 20% were vaccinated. Prior to BRH use, 62.4% reported COVID‐19 infection, compared to 11.2% after prophylaxis (*p* < 0.0001). Significant reductions were observed in both vaccinated (48.1% vs. 3.7%, *p* = 0.002) and unvaccinated individuals (66.3% vs. 13.3%, *p* < 0.0001). Prior infection did not significantly influence outcomes (*p* = 0.9248). Longer BRH use was associated with lower reinfection rates, decreasing from 33.3% (≤ 10 days) to 2.6% (≥ 30 days) (*p* = 0.003).

**Conclusions:**

BRH prophylaxis was associated with reduced reported COVID‐19 infection rates, independent of vaccination or prior infection, with greater benefit observed with longer use. Prospective controlled studies are required to confirm these findings.

## 1 Introduction

Severe acute respiratory syndrome coronavirus 2 (SARS‐CoV‐2) causes disease, coronavirus disease 2019 (COVID‐19), which presents an array of clinical severity from asymptomatic through severe disease, cytokine storm [CS], progressing to acute respiratory distress syndrome, coagulopathy, multiorgan dysfunction, shock, and death. The COVID‐19 pandemic has caused millions of human casualties and enormous financial damage to humanity. An enormous intellectual and financial resource has been thrown into finding a cure for COVID‐19. A Google Scholar search with the keyword *COVID‐19* shows 5,470,000 articles [as of February 09, 2026]. The final result regarding the treatment of this disease is deplorable. The World Health Organization (WHO) offers for outpatient treatment three antiviral preparations (Paxlovid, Remdesivir, and Molnupiravir) with a rather controversial effect [1, 2, 3, 4].

In a number of articles, we have advocated that the strategy to combat COVID‐19 is to block the SARS‐CoV‐2 from entering the cell and to inhibit the hyperactivated nucleotide‐binding oligomerization domain‐like receptor containing pyrin domain 3 (NLRP3) inflammasome (NLRP3‐I) which is a central mediator of severe COVID‐19 [5], causing the CS, with subsequent multiorgan damage and death [3, 4, 6, 7]. NLRP3‐I is involved in COVID‐19 respiratory manifestations, cardiovascular comorbidity, and neurological symptoms [8].

We therefore also believe that antiviral agents are limited in solving the problem, since there is no direct link between viral replication and hyperactivation of the NLRP3‐I [3, 4, 5, 8, 9, 10, 11, 12, 13].

Administration of antibodies blocking a given cytokine(s) is also doomed to failure because the cause of the CS, the hyperactivated NLRP3‐I, continues to produce hypercytokinemia [3, 4]. In a number of publications, we have demonstrated that blocking the NLRP3‐I is possible with high but safe doses of colchicine. If given on time, high doses of colchicine solve the CS problem [3, 4, 6, 7, 14, 15, 16, 17, 18, 19].

It is well known that SARS‐CoV‐2 uses the Angiotensin‐converting enzyme‐2 (ACE2), which acts as a receptor for the virus S protein for host cell entry [20].

The lungs are the most susceptible organ to SARS‐CoV‐2 infection, because about 85% of the cells that express ACE2 in the lungs are type 2 alveolar epithelial cells. ACE2 is also present in the throat, heart, kidneys, intestines, and other organs [21, 22].

The principal SARS‐CoV‐2 cell entry (via plasma membrane/early endocytosis) is related to the androgen‐regulated cell‐surface transmembrane protease serine subtype 2 (TMPRSS2). TMPRSS2 belongs to the very few trypsin‐like proteases expressed in the human respiratory tract. TMPRSS2 plays a role in the proteolytic activation and invasion of the human airway epithelium by influenza A [23, 24]. It is the major activating protease for the influenza B virus [25, 26], as well as SARS‐CoV and MERS viruses [27].

**Figure 1:**
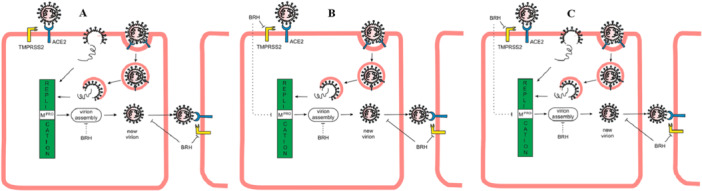
*Effect of BRH administered at different time intervals*. (A) The principal SARS‐CoV‐2 cell entry is related to TMPRSS2 (Entry via plasma membrane). The second way is through receptor‐mediated endocytosis (Endosomal entry). (B) When BRH is taken prophylactically, the main route of viral entry is blocked due to the inhibition of TMPRSS2. BRH could theoretically also affect viral replication by inhibiting Mpro, virion assembly, and spread. (C) When the disease occurs, the virus has already penetrated the cell, and the effectiveness of BRH is greatly reduced. However, some positive effects of BRH remain, with the blocking of virus spread and its anti‐inflammatory and mucolytic properties.

TMPRSS2 cleaves the spike protein, leading to the fusion of the viral and host membranes (Entry via plasma membrane/Non‐endocytosis/Early endocytosis/Cell surface entry). The binding of SARS‐CoV‐2 to ACE2 can result in uptake of virions into endosomes (Entry via endosomal pathway/Late endocytosis/Endosomal entry), where the S protein is activated by the pH‐dependent cysteine protease cathepsin B/L [28, 29, 30, 31] (Figure 1A). TMPRSS2 could act in both early and late endosome entry processes [20]. SARS‐CoV‐2 virions are enriched in the fragmented Golgi [32]. TMPRSS2 has been implicated in the regulation of the viral assembly in the Golgi apparatus and the release of the mature virus from the host plasma membrane [33, 34, 35]. TMPRSS2 is also responsible for the viral spread in the infected host. TMPRSS2 contributes to virus spread and immunopathology in the airways of murine models after coronavirus infection [20, 27, 36].

Other mechanisms of entry have also been described. Despite the existence of alternate receptors (e.g., neuropilin‐1, heparin sulfate) and entry mechanisms (antibody‐mediated endocytosis, transcytosis of virions to adjacent cells), the distribution of ACE2 and TMPRSS2 is a consistent predictor of SARS‐CoV‐2 tissue tropism [37, 38]. Three major cell types co‐express TMPRSS2 and ACE2—type II pneumocytes, ileal absorptive enterocytes, and nasal goblet secretory cells/nasal ciliated cells or both [39].

Since 1963, BRH, an over‐the‐counter, non‐invasive, effective, well‐tolerated medicine, with proven safety, affordable, available globally, and at low costs, has a long history of use in respiratory tract disorders. The mucolytic cough suppressant BRH is known for its effectiveness as an expectorant and mucolytic agent and has been shown to have anti‐inflammatory effects, thus reducing swelling and irritation in the respiratory tract [3, 40].

BRH is rapidly absorbed from the gastrointestinal tract; however, its first‐pass metabolism in the liver is high, and bioavailability when taken orally is only 20% [28]. BRH application by inhalation could circumvent the first‐pass effect. BRH is orally readily bioavailable, and thus, a more frequent and higher oral dose could have a stronger and longer‐term inactivation of the TMPRSS2 enzyme [28].

The active demethylated metabolite of BRH, ambroxol, is a potent inducer of surfactant synthesis in AT2 cells [41, 42]. BRH can prevent influenza infection [43], inhibiting TMPRSS2 [44].

BRH has a selective inhibitory effect on TMPRSS2, thus inhibiting the SARS‐CoV‐2 entry into the cell [20, 45]. Other authors suggest that BRH exerts its anti‐SARS‐CoV‐2 activity by inhibition of cathepsin rather than TMPRSS2 [46, 47], but in both cases, BRH blocked the entry of SARS‐CoV‐2 into cells. In addition, BRH demonstrated an antiviral effect by inhibiting the cysteine protease 3Clpro/Mpro, respectively disrupting the viral replication [48, 49].

BRH has been effective in controlling SARS‐CoV and MERS infections [26].

There is conflicting data about the role of BRH in the prevention and treatment of COVID‐19. Some small BRH trials suggest potential benefits, and others show no significant effect. Current studies on BRH use in COVID‐19 are limited to small sample sizes, in different settings, and for different durations. To date, no other long‐term prophylaxis study has been conducted. While meta‐analyses are limited to using studies with similar study designs, thus showing no convincing evidence for the effect of BRH as a prophylactic treatment for COVID‐19 [28, 30, 40, 44, 50, 51, 52, 53, 54].

In this study, we attempt to demonstrate the potent prophylactic effect of BRH, which prevents the viral penetration into host cells and protects against infection with COVID‐19 or promotes its mild course. We also hope that our hypothesis of the prophylactic role of BRH against COVID‐19 and influenza will attract attention and lead to other studies, including randomized, blinded clinical trials.

### 1.1 Aim of the Study

The main purpose of the study was to record the patient‐reported outcome (covid infections vs no‐covid infection) among people, using BRH as a prophylactic agent during the peak waves of contagion between 2021 and 2022.

For context, at the end of 2020, an official announcement by Prof. Dr. Vanyo Mitev within the faculty of Medicine, Medical University of Sofia, supported the use of BRH as a way to prohibit SARS‐CoV‐2 entry into the cells and prevent infection. This call was reflected in the media in 2021 https://www.bgonair.bg/a/36-sutreshen-blok/246992-akad-mitev-lekuvayte-kovid-19-s-bromheksin-i-kolhitsin, with many of the clinics and attending doctors heeding it.

This study is classified as a non‐interventional clinical study (i.e., a study i.e. not a clinical trial). All materials were approved by the institutional ethics committee (decision number:12/17.062024).

## 2 Materials and Methods

This is a retrospective, observational, questionnaire‐based study of patient and staff experiences with BRH prophylactic treatment during the COVID‐19 peaks in contagion in the outbreaks during 2021 and 2022.

### 2.1 Recruitment and Procedure

The study focused on both patients and staff within the clinics of the Medical University of Sofia, Bulgaria, between January 2021 and December 2022. Patients attending routine check‐ups once yearly were given instructions for BRH prophylactic use during the pandemic. At attendance, previous exposure to COVID was ascertained, and at the end of 2022, patients were asked to fill in a questionnaire survey.

For staff, a standard operating procedure (SOP) was distributed on the prophylactic use of BRH when dealing with patients and medical students. In January 2021, staff reported on previous COVID‐19 infections, and at the end of 2022, filled out the same questionnaire as patients.

All subjects were given the same standardized questionnaire, where we recorded overall medication intake during COVID‐19, specific sections on whether and how BRH was applied prophylactically, the daily number of tablets, and the duration of prophylactic use in days. A question asking if the subjects had had a COVID‐19 diagnosis confirmed during this time period, and the severity of symptoms. A minimum of 10 days of continuous intake of BRH tablets was considered “prophylactic”. Further questions on vaccination status were included, as well as “post‐COVID‐19” symptoms.

Data on Height, weight, and age were also collected, allowing for BMI calculation.

### 2.2 Outcomes

The main outcome of interest was defined as “confirmed subsequent infection with COVID‐19”, after BRH addition as a prophylactic agent.

Secondary outcomes included severity of symptoms and “post‐ COVID‐19” symptoms for the subsequent 30 days. A total of 13 post‐ COVID‐19 symptoms were included in the questionnaire:

1. Fatigue, or unexplained tiredness
2. Constant fatigue, despite rest
3. Finding it “difficult to breathe”
4. Chest discomfort (such as tightness, or as if pricked by a needle)
5. Coughing fits
6. Increased heart rate without exertion
7. Increased body temperature
8. Muscle or joint pain
9. Headache
10. Impaired cognitive reasoning, function, or concentration abilities
11. Difficulty sleeping/lower quality of sleep
12. Loss of sense of smell
13. Hair‐loss

### 2.3 Statistical Analysis

Both groups were analyzed together as “responders.”

Patient responses were recorded and coded into Excel for descriptive statistical analysis with MedCalc Statistical Software version 22.0.0 (MedCalc Software bvba, Ostend, Belgium; http://www.medcalc.org; 2024).

Paired samples *T*‐test was used to ascertain differences in COVID‐19 infection before and after BRH prophylactic treatment in all patients. Further stratification on infection rate was done for “non‐vaccinated” and “vaccinated” individuals. An independent samples t‐test was used to analyze the mean secondary infection rate among these individuals.

Patients were subject to further subgroup analysis, based on daily dose and duration of prophylactic treatment: no more than 10 days; no more than 20 days; no more than 30 days; continuous intake for over 30 days. This allowed for coding as a categorical variable, avoiding responses such as “approximately 2 weeks”, which were observed in previous versions of the questionnaire. One‐way ANOVA was used to analyze subgroups.

A multivariable logistic regression analysis was performed to evaluate factors associated with COVID‐19 reinfection. Age, BMI, vaccination status, previous COVID‐19 infection, number of comorbidities, and duration of bromhexine intake were included as independent variables. Adjusted odds ratios (ORs) with 95% confidence intervals (CIs) were calculated. Model discrimination was assessed using the area under the receiver operating characteristic curve (AUC), and goodness of fit was evaluated using the Hosmer–Lemeshow test. Statistical significance was defined as *p* < 0.05.

### 2.4 Data Collection and Curation

All responses were pseudonymized, where all identifiable information was removed. Assessors were given a coding system to code responders based on the location where the questionnaire was administered (letter‐based—A–Z) and the sequential entry into the dataset (number‐based – 1–1000), resulting in patient identifiers such as C003. A pragmatic cohort approach was utilized to derive the final sample size, due to the limited nature of COVID‐19 contagion peaks.

## 3 Results

### 3.1 Baseline Responder Characteristics

A total of 376 responses were received, with 125 responses of confirmed cases of prophylactic usage of BRH extracted from them and considered eligible. Mean and median ages for the 125 included responders were 56.46 and 58 years, respectively. The mean BMI of responders was 25.59. Duration of BRH intake varied among responders, as well as daily doses (Table 1). The majority of responders reported taking BRH prophylactically at least 20 to 30 days per year (2021 and 2022), with the mean duration showing 47.52 days, and the median 30 days. Detailed prophylactic regimens are available in Table 1. Only 20% of responders reported being vaccinated.

**Table 1:** Baseline characteristics of patients.

	Mean (95% CI)	Median	Lowest	Highest
Age	56.46 years (53.23–59.69)	58 years	16 years	98 years
BMI	25.59 (24.53–26.64)	24.25	14.52	45.91
Number of Comorbidities	1.33 (1.15–1.52)	1	1	4
Duration of bromehexine intake	47.51 days (38.04–56.99)	30 days	10 days	365 days
Daily bromhexine intake	41.15 mg (37.82–44.48)	48 mg	8 mg	128 mg
Vaccination rate	20%			

### 3.2 Reported Comorbidities

The number of comorbidities reported was low, with a median of 1 comorbidity per patient. 64 (51.2%) responders reported they had no comorbidities, and among the remaining 61 responders, the highest recorded comorbidity was hypertension with 49 counts (Figure 1). Among responders, 35.2% (*n* = 47) had one comorbidity, 6.4% (*n* = 8) had 2; 4% (*n* = 5) had 3 comorbidities, and only 1 patient reported four concomitant comorbidities.

Responders varied greatly in age, with the highest number of people observable in the age brackets < 40 years and > 75 years (Table 2). Elderly patients (defined as patients 65%) showed a higher share of comorbidities, with patients aged 75% having a comorbidity prevalence of 12.8%. Another observable trend is the increase in BMI. The highest recorded BMI was for patients aged 65–69 years, and in general, as age increases, so do the underlying risk factors such as increased BMI and presence of comorbidities. This confirms that elderly patients are at higher risk of COVID‐19 mortality. Despite the low average BMI recorded for the patient population, 23 cases of obesity (defined as a BMI > 30) were recorded, corresponding to a prevalence of 18.4%.

**Table 2:** Age distribution among responders.

Age group	Number	Average BMI	Number who reported comorbidities	% In group with comorbidity (% from total)
< 40	24 people	21.96	5 people	20.83% (4%)
40–44	7 people	21.37	1 person	14.28% (0.8%)
45–49	9 people	25.17	1 person	11.11% (0.8%)
50–54	16 people	26.46	7 people	43.75% (5.6%)
55–59	9 people	27.89	7 people	77.77% (5.6%)
60–64	11 people	24.94	4 people	36.36% (3.2%)
65–69	15 people	29.39	8 people	53.33% (6.4%)
70–74	11 people	26.33	8 people	72.72% (6.4%)
75+	23 people	26.14	16 people	69.56% (12.8%)
Total	**125 people**	**25.59**	**57 people**	**45.6%**

### 3.3 COVID‐19 Status

At the time of recruitment, 63.41% (*n* = 78) reported a previously confirmed COVID‐19 diagnosis. At follow‐up and upon BRH inclusion, the percentage with a subsequent infection fell to 11.2% (*n* = 11). This represents a statistically significant difference of 52.28% in subsequent infection cases (*p* < 0.0001).

Vaccination coverage was low in the sample, with 27 individuals reporting being vaccinated (21.6%). Out of those, 12 responders confirmed they had been vaccinated prior to their first infection (9.6%), with the corresponding 15 responders being vaccinated after their first COVID‐19 infection (12%).

Among the entirety of vaccinated individuals, the probability of COVID‐19 infection, prior to BRH prophylactic intake, was 48.15%, with 13 total cases of COVID‐19 out of 27 vaccinated individuals. During the follow‐up interview, only 1 patient reported having a secondary infection (3.7%), corresponding to a 44.45% decrease in re‐infection likelihood (*p* = 0.002). It is worth noting that this single patient had a BMI of 42, 3 comorbidities, and had his vaccine after his first COVID‐19 infection, thus can be considered a very “high‐risk” patient.

Among unvaccinated individuals (*n* = 98), the infection rate prior to BRH infection was reported at 66.32% (*n* = 65). Upon BRH inclusion, this percentage went down to 13.26% (*n* = 13), representing a statistically significant difference of −53.06% (*p* < 0.0001) (Table 3).

**Table 3:** Infection percentages among vaccinated and unvaccinated responders.

	Infection % prior to BRH	Infection % after BRH inclusion
Vaccinated (*n* = 27)	48.15% (*n* = 13)	3.7% (*n* = 1)
Unvaccinated (*n* = 98)	66.32% (*n* = 65)	13.26% (*n* = 13)
Overall (*n* = 125)	62.4% (*n* = 78)	11.2% (*n* = 14)

To differentiate the effect‐estimate of antibodies on infection likelihood, two separate groups were further stratified—unvaccinated with a prior infection (*n* = 65), vs unvaccinated without a prior infection (*n* = 33). Among the 65 non‐vaccinated with possible antibodies, receiving BRH prophylactic treatment, the rate of subsequent re‐infection was 13.8% (*n* = 9). Among the 33 unvaccinated without antibodies, the subsequent infection rate was 12.2% (*n* = 4). The difference was statistically insignificant (*p* = 0.9248) between the two groups.

### 3.4 Duration of Prophylactic Treatment and Outcomes

One‐way ANOVA analysis confirmed that duration of prophylactic treatment is related to improved outcomes and lower re‐infection likelihood. Patients taking BRH for more than 30 days had a significantly lower re‐infection rate than patients taking it for no more than 10 days. As the duration of prophylactic intake of BRH increased, so did the likelihood of infection correspondingly fall among these patients. Although no statistical significance was confirmed for the group taking BRH for no longer than 20 days, their reinfection rate was still lower than group 1, and higher than group 3 (difference of −19.04%, and +10.44%, respectively)

Within the subgroup of 21 patients taking BRH for more than a month, 13 were unvaccinated (with 1 having a prior infection), while 8 had been vaccinated, noting a positive effect, which lacked the sample size to confirm this trend statistically. (Table 4).

**Table 4:** ANOVA analysis of reinfection likelihood, stratified by duration of prophylactic treatment.

Factor		N	Mean reinfection rate	SD	Different (*p* < 0.05) than factor
1.	No more than 10 days	18	0.3333 (33.33%)	0.4851	3, 4
2.	No more than 20 days	42	0.1429 (14.29%)	0.3542	
3.	No more than 30 days	26	0.03846 (3.84%)	0.1961	1
4.	30 days or more	39	0.02564 (2.56%)	0.1601	1
*F*‐ratio		4.927			
Significance level		*p* = 0.003			
Levene statistic		20.202			
Significance		*p* < 0.001			

### 3.5 Logistic Regression Analysis

Multivariable logistic regression was performed to evaluate predictors of COVID‐19 reinfection while adjusting for potential confounders. The overall model was statistically significant (χ²=23.99, *p* = 0.0005) and demonstrated good discrimination (AUC = 0.804). The number of comorbidities was a significant predictor of reinfection (OR = 2.90, 95% CI 1.47–5.73, *p* = 0.002), while age was inversely associated with reinfection risk (OR = 0.95 per year, 95% CI 0.91–0.99, *p* = 0.0099). Duration of bromhexine use showed a non‐significant trend toward reduced reinfection risk (OR = 0.89, 95% CI 0.77–1.05, *p* = 0.16). Other variables, including BMI, vaccination status, and prior infection, were not statistically significant in the adjusted model (Table 5).

**Table 5:** Multivariable logistic regression analysis of predictors of COVID‐19 reinfection (*n* = 125).

Variable	Coefficient (β)	SE	Adjusted OR	95% CI for OR	*p* value
Age (years)	−0.0544	0.0211	0.9470	0.9086–0.9870	0.0099
BMI (kg/m²)	0.0666	0.0570	1.0689	0.9560–1.1952	0.2422
Previous COVID‐19 infection (yes vs no)	−0.5161	1.0821	0.5968	0.0716–4.9763	0.6334
Number of comorbidities	1.0656	0.3472	2.9027	1.4697–5.7327	0.0021
Duration of bromhexine use (days)	−0.1116	0.0796	0.8944	0.7653–1.0454	0.1608

*Note:* Model performance: likelihood ratio test *p* = 0.0005; Hosmer–Lemeshow *χ*² = 8.4116, df = 6, *p* = 0.3943; AUC = 0.804 (95% CI 0.720–0.872); overall classification accuracy = 80.17%.

Abbreviations: BMI, body mass index; CI, confidence interval; OR, odds ratio; SE, standard error.

### 3.6 Analysis of Secondary Outcomes

Out of all 125 responders, 13 replied they haven't had post‐COVID‐19 symptoms, 104 left that part of the questionnaire blank, and only 8 filled‐in questionnaires confirmed the presence of post‐ COVID‐19 symptoms (3 male, 5 female). These 8 patients had the following characteristics: Average BMI 25.56, all had been vaccinated prior to their first infection, no infection after initiating BRH therapy, none of them had been hospitalized due to COVID‐19 infection, none of them were active or previous smokers, and only 3 had a comorbidity, with that comorbidity being Hypertension for all 3. On average, the number of post‐ COVID‐19 symptoms was 5; however, there didn't seem to be a discernible connection between the severity of their post‐ COVID‐19 experience and any recognized risk factors (Table 2). All 8 responders had a duration of BRH prophylaxis less than 20 days.

## 4 Discussion

### 4.1 Game of Time

While for colchicine the dosage is important—just the right dose [game of dosage], for BRH we hypothesized that not the dose, but rather the duration of administration is decisive – just the right duration [game of time].

When predicting the effectiveness of BRH, the question of when and how it should be applied comes to the forefront. This stems from the fact that the peak of SARS‐CoV‐2 load is reached before or simultaneously with the onset of symptoms. Thus, when the first symptoms appear, the viral load is already at its maximum [55].

Therefore, even if the treatment with BRH starts from the first day of symptom onset, the virus has already penetrated the cells, significantly reducing the efficacy of BRH, especially if not inhaled [3, 6, 14].

The second issue of note is what precisely is the optimal duration of prophylactic administration, that can reduce the likelihood of re‐infection. In our study, the median duration of BRH intake was 30 days. The one‐way ANOVA analysis clearly indicated that increasing the duration of prophylactic intake results in lower re‐infection likelihood, which was also evident in the logistic regression analysis. Increasing the duration of intake was associated with lowered odds of reinfection. Although not statistically significant, this is a signal of potential benefit that should be further investigated.

A further beneficial effect was observed for vaccinated individuals. Although both sets of individuals, vaccinated and unvaccinated, experienced lower reinfection likelihood, the presence of vaccine‐induced antibodies seemed to enhance the beneficial effect. Our data indicates that individuals with antibodies who maintain a minimum of 30 days of intake have virtually negligible odds of reinfection. These findings are consistent with what is known about the innate immune response and antibody dynamics during COVID‐19 infection. Specific antibodies to the S‐glycoprotein of SARS‐CoV‐2 result only after exposure, where IgM antibodies that form within a week of infection last only 12 weeks, while IgG antibodies last longer [56]. A functioning immune response has also humoral components, whereby even if small viral quantities manage to enter the cells, they are swiftly dealt with. Our study did not account for the number of contacts (i.e., their social activity) these responders have had during the study period. The logistic regression possibly accounts for some of these confounders since increasing age was negatively related to reinfection likelihood, which could reflect either the BRH benefit or reduced number of contacts as age grows. Nonetheless, this presents a considerable limitation to the study. Other limitations can be outlined in the way the study was conducted. Patient‐reported outcomes suffer from a high‐risk of bias and are prone to missing information, as evidenced by the lack of information on post‐COVID‐19 symptoms. There was no special selection or invitation for the patients and medical staff. Information for the use of bromhexine as a “protective” agent was distributed in front of the offices and among the medical staff. Those who wanted to participate fulfilled the questionnaire themselves, which resulted in a higher proportion of invalid questionnaires (251 vs 125). Despite this, it is worth noting that the presence of post‐COVID‐19 symptoms in our sample did not show any connection to the established risk factors, such as age, sex, or the presence of comorbidities [57]. Unfortunately, inconsistent reporting on height and weight prevented us from conducting BMI stratification of groups.

### 4.2 Benefits of Prophylaxis With BRH

#### 4.2.1 Нypothesis

BRH is most effective when taken prophylactically, because it blocks in advance the entrance of the SARS‐CoV‐2 to the cell (Figure 1B). Prevention is best done before the next COVID‐19 wave with BRH tablets for a minimum of 1 month.

**Reasoning:** When the virus has penetrated the cell, the chief concern is what will be the reaction of the NLRP3‐I. Complications can occur only with its hyperreaction, the reasons for which are unknown. In NLRP3‐I hyperreactivity, high doses of colchicine resolve the problem. In our sample, patients who got sick had a mild disease, which lends credence to the hypothesis but requires further testing.

### 4.3 Benefits of Post‐Exposure Prophylaxis With BRH

#### 4.3.1 Нypothesis

When someone has been in contact with an infected or sick person, BRH inhalations should be started immediately. If the BRH inhalations are undertaken immediately after contact, the probability that the infection does not progress is high. The timing and the mode of administration are crucial [3]. The outcome of the competition, whether the virus will enter the cell before BRH is able to block TMPRSS2, will determine whether or not an infected person will become ill.

#### 4.3.2 Reasoning

Studies using BRH as post‐exposure prophylaxis show that the incidence of symptomatic COVID‐19 was significantly lower in individuals who received BRH than in those who received the placebo, with a relative risk reduction of 50%. The hospitalization rate, death, and medication side effects did not vary significantly between the BRH and placebo arms [50]. A lower frequency of symptomatic SARS‐CoV‐2 infection among medical personnel taking BRH (8 mg three times a day) compared to controls was reported [58]. The small sample size was the main limitation of that study, but we also observed broader indications that this trend is true. A secondary aspect worth investigating is whether the results with inhaled BRH would be different.

### 4.4 Benefits of Using BRH as a Remedy

#### 4.4.1 Нypothesis

When the first symptoms of COVID‐19 appear, the virus has already entered the cell. As we noted above, the viral load is already at its maximum [55]. This greatly limits the prophylactic effect of BRH. However, as TMPRSS2 is responsible for the viral assembly and spread [20, 27, 33, 34, 35, 36, 37], the immediate inhalation of BRH may help limit contamination. A probable effect on viral replication (inhibition of Mpro) is also possible (Figure 1C) [48, 49].

In addition, BRH is an effective expectorant and mucolytic agent having anti‐inflammatory effects, thus reducing swelling and irritation in the respiratory tract, helping to relieve cough, lassitude, congestion, and dyspnea specific to COVID‐19 [40]. These cardinal respiratory symptoms were remarkably less in COVID‐19 patients who received BRH treatment than in the standard group [30].

All this, plus its very low side effects, should be strong arguments for the use of BRH in the treatment regimen for COVID‐19. However, it must be strongly emphasized that after the penetration of SARS‐CoV‐2 into the cell, it is not the replication of the virus that leads to disease aggravation, but the abnormal reaction of the NLRP3‐I.

## 5 Results

Several trials have been conducted to determine BRH efficacy; however, its usefulness remains controversial [28, 30, 40, 44, 50, 51, 52, 53, 54].

In our experience, administration of inhaled BRH is beneficial in both outpatient and inpatient settings [3]. This will be more or less effective, but in no case decisive, to avoid possible complications. As we have already noted, only inhibition of the hyperactivated NLRP3‐I can save the patient from CS, multiorgan damage, and death. BRH's mechanism of action indicates that benefits should be more pronounced when given prophylactically over the course of a month during the peak of another COVID‐19 wave or by inhalation as **a** post‐exposure control measure.

Many hopes were placed on the TMPRSS2 inhibitor Camostat Mesylate (CM), previously used for the treatment of pancreatitis and reflux oesophagitis, now for the treatment of COVID‐19 [20]. CM trials have focused on COVID‐19 treatment rather than prevention and a large number of prestigious clinical trials have been launched to investigate its effectiveness: Denmark (CamoCO‐19, NCT04321096), USA (RECOVER, NCT04470544, NCT04353284, NCT04524663, NCT04374019), UK (NCT04455815), Mexico (NCT04530617), Israel (COSTA, NCT04355052), Germany (NCT04338906), South Korea (NCT04521296) and Japan (NCT04451083).

Against the backdrop of high expectations, the results are discouraging [59, 60, 61, 62]. This oversight could have been predicted based on detailed molecule analysis, because CM has no way to block the SARS‐CoV‐2 from entering the cell, since by the time it is applied, the virus has already entered. Inhibiting TMPRSS2 after the virus has long since entered the cell, replicated, and infected other cells is doomed to failure [63, 64, 65]. Compared to CM, BRH can accumulate in pulmonary and bronchial epithelial cells [66]. BRH demonstrated a greater inhibitory effect than CM [67], and has fewer side effects, is cheaper, and more accessible.

Very recently, cases of elderly women with severe comorbidities were published, who, after long‐term prophylaxis with BHH, avoided infection with COVID‐19, despite their immediate families becoming seriously ill [68].

### 5.1 COVID‐19‐Influenza Connection

TMPRSS2 blockade offers advantages over other viral entry inhibitors, because it serves as a major cofactor rather than cathepsins for SARS‑CoV‑2 cell entry and it is the major hemagglutinin‑activating protease of influenza viruses [64, 69]. This may explain the cases of influenza virus and SARS‐CoV‐2 co‐infection [70]. On the other hand, the major role of TMPRSS2 in the regulation of SARS‑CoV‑2/influenza entry into the cell suggests successful prophylaxis after its preventive inhibition. In addition, as the COVID‐19/influenza complications are due to the hyperreaction of the same inflammasome (NLRP3‐I), this suggests the same method of treatment [63, 64, 71]. Prophylactic administration of BRH during the 2024/2025 influenza epidemic in Bulgaria led to a 3.5‐fold reduction in morbidity [65] and, accordingly, to great economic efficiency [72]. Similarly, during the 2025/2026 flu epidemic in Bulgaria, the prophylactic use of BRH, based on preliminary data from over 1000 participants, yielded overlapping results [73]. Thus, after additional randomized controlled trials, BRH could be a cost‐effective alternative when used off‐label for the prophylaxis of influenza A and B and COVID‐19.

The mucolytic agents BRH and its derivative ambroxol have also shown potential benefits in the treatment of various extrapulmonary disorders, raising the issue of the use of these drugs in multisystem disorders [74, 75]. Their good safety profile, low cost, availability, and affordability are good reasons to explore their extrapulmonary use as well.

All of these factors point us to believe BRH could be a serious candidate for drug repurposing and warrant further investigation. As such, we hope this study will encourage broader scientific discussion.

## 6 Conclusions

BRH significantly reduces the likelihood of COVID‐19 infection, with this effectiveness being more pronounced in individuals with antibodies. Our study suggests that vaccines and BRH have complementary mechanisms that allow for a higher degree of protection in 2 possible ways: firstly, BRH inhibits viral cell entry; secondly, if the virus still manages to gain entry, patients with S‐glycoprotein‐specific antibodies are able to swiftly deal with the infection before peak cell load.

Based on our experience since 2020, we propose it is appropriate to test the following possibilities: mass prophylaxis with BRH/ambroxol during another COVID‐19 wave; when in contact with a sick or infectious person ‐ immediate inhalations with BRH/ambroxol; in case of illness ‐ high doses of colchicine and inhaled BRH/ambroxol. If confirmed, this could help cut hospital admissions and reduce treatment to the outpatient setting. Prevention with BRH is of particular importance for the most at‐risk groups ‐ adults with high BMI and pronounced comorbidity.

Just as our results with the use of high doses of colchicine were recently confirmed by a randomized double‐blind clinical trial [76], we hope to provoke such studies on the prophylactic role of BRH for COVID‐19 and influenza.

## Author Contributions

**Vanyo Mitev:** conceptualization, methodology, data curation, investigation, validation, formal analysis, supervision, funding acquisition, visualization, project administration, resources writing, original draft. writing review and editing. **Tsanko Mondeshki:** investigation, methodology, validation resources. **Ani Miteva:** methodology, visualization, writing review and editing, formal analysis, data curation. **Konstantin Tachkov:** writing review and editing, software, formal analysis. **Violeta Dimitrova:** investigation, validation, formal analysis, data curation.

## Conflicts of Interest

The authors declare no conflicts of interest.

## Data Availability

All data is available upon request from the authors. Authors give permission to reproduce material.
